# Targeting molecular resistance in castration-resistant prostate cancer

**DOI:** 10.1186/s12916-015-0457-6

**Published:** 2015-09-01

**Authors:** Thenappan Chandrasekar, Joy C. Yang, Allen C. Gao, Christopher P. Evans

**Affiliations:** Department of Urology, University of California, Davis, USA; 4860 Y Street, Suite 3500, Sacramento, CA 95817 USA

**Keywords:** Castration-resistant, Disease progression, Drug resistance, Prostatic neoplasms

## Abstract

Multiple mechanisms of resistance contribute to the inevitable progression of hormone-sensitive prostate cancer to castration-resistant prostate cancer (CRPC). Currently approved therapies for CRPC include systemic chemotherapy (docetaxel and cabazitaxel) and agents targeting the resistance pathways leading to CRPC, including enzalutamide and abiraterone. While there is significant survival benefit, primary and secondary resistance to these therapies develops rapidly. Up to one-third of patients have primary resistance to enzalutamide and abiraterone; the remaining patients eventually progress on treatment. Understanding the mechanisms of resistance resulting in progression as well as identifying new targetable pathways remains the focus of current prostate cancer research. We review current knowledge of mechanisms of resistance to the currently approved treatments, development of adjunctive therapies, and identification of new pathways being targeted for therapeutic purposes.

## Background

Prostate adenocarcinoma is the second-leading cause of cancer-related deaths and is the most commonly diagnosed non-cutaneous malignancy in men [1, 2]. Despite the focus on screening and early detection of prostate cancer, approximately 20 % of men continue to present with advanced or metastatic disease [3], and there were more than 29,000 prostate cancer-related deaths in the United States in 2014 alone [1].

The androgen axis is an important component of prostate cancer physiology. The androgen receptor (AR) is a 110 kDa nuclear receptor encoded by the *AR* gene, which is on Xq11-12 and has eight exons. It is part of a family that includes the mineralocorticoid, glucocorticoid, estrogen, and progesterone receptors. It has four functional motifs – the amino-terminal domain (N-terminal domain, NTD), DNA binding domain, hinge region, and ligand-binding domain (LBD) [4, 5]. It is bound by heat-shock proteins in the inactive state in the cytoplasm, until androgen binding to the LBD causes a conformational change that leads to heat-shock protein disassociation, homodimerization of the receptor, and subsequent nuclear translocation. In the nucleus, it binds to androgen-response elements in the promoter regions of AR-regulated genes [6, 7]. Androgens, specifically testosterone, the primary circulating androgen produced mainly in the Leydig cells in the testis and minimally in the adrenal cortex, and dihydrotestosterone (DHT), are the major ligands for AR. DHT, which is formed by 5α-reductase activity on testosterone within the cytoplasm, is the main functionally active ligand in the prostate microenvironment, and has a 5-fold higher affinity for the LBD of AR than testosterone [8–10].

In patients who are diagnosed with or progress to advanced or metastatic prostate cancer, the treatment standard is currently androgen-deprivation therapy (ADT). First described by Huggins and Hodges in a dog model [11], ADT now is achieved through either surgical (bilateral orchiectomy) or medical castration. Medical castration utilizes different classes of agents, including LHRH agonists, LHRH antagonists, and anti-androgens. However, despite an initial benefit, the majority of patients will progress to castration-resistant disease within 2–3 years of initiation [12].

Castration-resistant prostate cancer (CRPC), previously called hormone-resistant prostate cancer, is defined as progression of disease, either clinical or biochemical, in the presence of castrate levels of circulating testosterone (<50 ng/dL) [13, 14]. The understanding that the androgen axis continues to play an important role in CRPC has led to further research and identification of therapeutic modalities for this patient population.

**Fig. 1:**
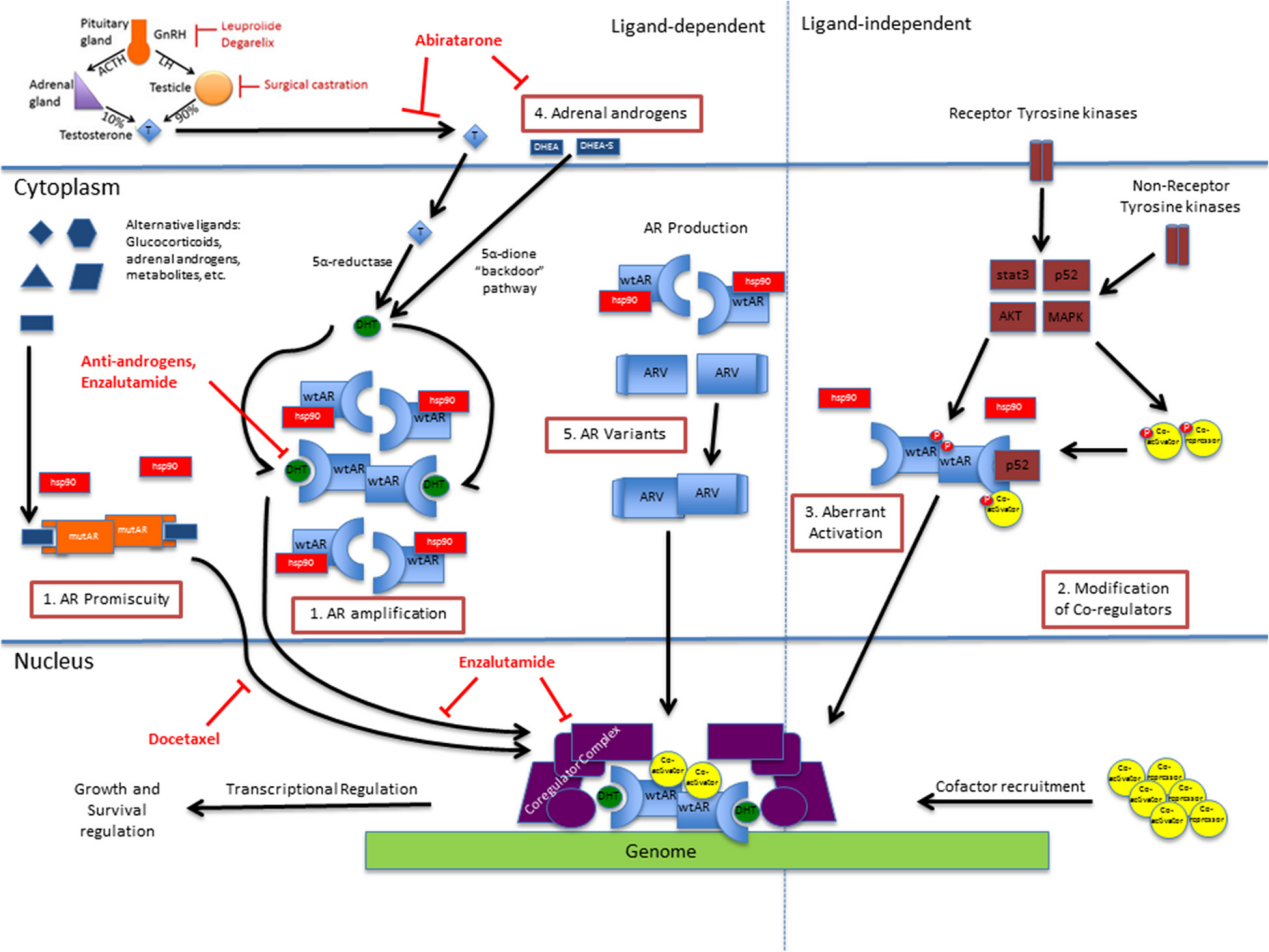
Androgen receptor-dependent mechanisms of resistance in hormone-naïve prostate cancer leading to castration-resistance, and role of current FDA-approved therapies. wtAR, Wild-type androgen receptor; ARV, Androgen receptor variant; mutAR, Mutated androgen receptor; T, Testosterone; DHT, Dihydrotestosterone

The mechanisms by which hormone-sensitive prostate cancer progresses to CRPC have been studied extensively. They can be subcategorized into five general categories – AR amplification and mutation, co-activator and co-repressor modifications, aberrant activation/post-translational modification, altered steroidogenesis, and AR splice variants. AR amplification, which allows continued androgen-axis activation in the presence of low levels of androgens in the prostate microenvironment, is found in 30–80 % of CRPC cell lines [15, 16]. AR point mutations lead to increased AR activity in the same microenvironment, but also broaden the ligand pool to which AR responds, including non-androgenic steroids [17–23]. Over 150 molecules have been identified as co-activators and co-repressors to the AR, and mutations in various components in the coregulator complex have been shown to improve androgen-stimulated AR activation and lead to progression of disease [24–27]. Aberrant activation encompasses pathways that activate AR in a ligand-independent manner [28–30]. Alterations in the steroidogenesis pathways allow prostate cancer cells to bypass testosterone, and utilize adrenal androgens to generate the functionally more potent DHT via the 5α-dione pathway [31–35]. Androgen receptor splice variants (ARV), which will be addressed in more detail later, are constitutively active modifications of the wild type AR. Figure 1 reviews the androgen axis and currently approved therapies.

The treatment options for CRPC continue to grow. Docetaxel, a well-known chemotherapeutic agent utilized in the treatment of multiple malignancies, is a current standard of care for this patient population, and for a long time was the only option for treatment. However, with research focusing on the mechanisms of progression to CRPC, newer agents have been identified that target those pathways specifically. The two approved medications, enzalutamide (MDV, Xtandi) and abiraterone acetate (Zytiga), work as an AR signaling inhibitor and a CYP17A1 steroidogenesis inhibitor, respectively. However, despite the survival benefit they provide, the disease will continue to progress.

Primary resistance is a problem in and of itself. Not all treatment-naïve patients are responsive to their administration. Approximately one third of patients treated with abiraterone in the COU-AA-301 trial had radiographic progression at 3 months [36] and one fourth of patients treated with enzalutamide in the AFFIRM trial had radiographic progression at 3 months [37], thereby demonstrating primary resistance. Of the patients who did respond, survival benefit was 3.9 months and 4.8 months, respectively; secondary progression eventually occurred by 24 months in virtually all patients despite initial benefit.

In an effort to understand the methods of targeting resistance in metastatic CRPC, we will review the current treatment modalities and the resistance mechanisms that have been identified.

## Current CRPC treatment modalities and target areas for new therapies

### Docetaxel and cabazitaxel

Docetaxel chemotherapy is a current standard of care for patients with CRPC, based on the SWOG 9916 and TAX327 trials, which demonstrated a 3-month survival advantage of docetaxel therapy over mitoxantrone [38, 39]. Until recently, it was the primary option for CRPC patients, but with the approval of abiraterone and enzalutamide, it is often not the first line therapy of choice. However, the recent “ChemoHormonal therapy versus androgen ablation randomized trial for extensive disease in prostate cancer” (CHAARTED) trial, which was a phase III randomized trial comparing docetaxel and ADT vs ADT alone in hormone-naïve prostate cancer, has now demonstrated the role for docetaxel as an initial treatment option for hormone-naïve prostate cancer in conjunction with ADT, providing a 17-month survival advantage [40]. This benefit was seen only in patients with high volume or visceral metastases. At the American Society of Clinical Oncology 2015 conference, James et al. presented the initial results of the “Systemic Therapy in Advancing or Metastatic Prostate Cancer: Evaluation of Drug Efficacy: A Multi-Stage Multi-Arm Randomised Controlled Trial” (STAMPEDE). In that trial, men with high-risk locally advanced or metastatic prostate cancer were randomized to four arms – hormone therapy, hormone therapy + docetaxel, hormone therapy + zoledronic acid, or hormone therapy + docetaxel + zoledronic acid. At completion, additional docetaxel added a 10-month survival benefit over hormone therapy alone in this patient population, which supports the findings of the CHAARTED trial [41].

Docetaxel is an anti-mitotic chemotherapeutic agent that works by binding the β subunit of tubulin in microtubules, thereby stabilizing the entire microtubule, preventing depolymerization and inhibiting mitosis [42–44], which induces apoptosis. It is a well-studied chemotherapeutic agent, and there is a great deal of literature on the resistance mechanisms towards docetaxel. Drug-efflux enables resistance to docetaxel in multiple different malignancies, including CRPC – multi-drug resistant proteins include p-glycoprotein, multi-drug resistant protein 1, and breast cancer resistance protein [45, 46]. Additionally, upregulation of class III β-tubulin isoform in docetaxel-resistant cell lines has been identified, since this isoform results in less stable microtubules; inhibiting this isoform restored docetaxel sensitivity [47–49]. However, targeting these mechanisms is not widely applicable to CRPC.

Multiple prostate cancer-specific pathways have been identified to contribute to docetaxel resistance, though many have not resulted in any clinically targetable treatments. A few of these pathways, however, are worth noting. Docetaxel resistance has been linked to apoptosis pathways, specifically upregulation of p53, an important cell cycle regulator often found overexpressed in prostate cancer, and activation of PAR1, which limits docetaxel-induced apoptosis through NF-κB activation [50–52]. Docetaxel’s antimitotic activity itself induces survival pathways in prostate cancer cells, such as the c-Jun N-terminal kinase, which in turn leads to activation of many transcription factors including STAT-1, STAT-3, and NF-κB; knockdown models of these transcription factors have been shown to be more docetaxel sensitive [50, 53]. Over-expression of chaperone proteins such as HSP27, HSP90, and clusterin have also been demonstrated to contribute to docetaxel resistance. A second-generation antisense drug, OGX-011, which inhibited clusterin secretion, was tested in conjunction with docetaxel in phase III trials, but did not meet its primary endpoint [54–56].

In an effort to target docetaxel resistance, our lab identified >1600 genes in taxane-resistant C42B cells that had altered expression. Of the 52 % that were upregulated, we identified a member of the ATP-binding cassette transporter family ABCB1 that was very highly upregulated in taxane-resistant C42B cells but essentially unchanged in taxane-sensitive cells. Inhibition of ABCB1 with ABCB1 shRNA resensitized taxane-resistant C42B and DU-145 cells to docetaxel and enhanced apoptotic cell death [52]. This was then confirmed with the use of Elacridar, an ABCB1 inhibitor, in both cell lines. Apigenen, a naturally occurring member in the flavone family that was originally demonstrated to resensitize cells to docetaxel chemotherapy [57], was found in our study to downregulate ABCB1 expression in a dose-dependent manner and reverse docetaxel resistance [52].

Cabazitaxel, a novel taxane approved for use in patients with CRPC who have failed docetaxel chemotherapy, is gaining traction in the treatment of CRPC. The TROPIC clinical trial identified cabazitaxel as having a 2.4-month survival benefit compared to mitoxantrone in patients with metastatic CRPC who had progressed on docetaxel [58]. Besides the clinical importance of this result, it also suggested that cabazitaxel had a novel mechanism of action [59] and did not share the same resistance mechanisms. Indeed, cabazitaxel was specifically selected for its poor affinity for p-glycoprotein 1 due the latter’s noted role in docetaxel resistance [60, 61].

### Abiraterone acetate

Abiraterone acetate (Zytiga) is an irreversible inhibitor of CYP17A1 that is structurally similar to pregnenolone. CYP17A1 has two consecutive enzymatic functions in the steroidogenesis pathway that contributes to the conversion of pregnenolone to DHT, and its loss causes significant loss of androgen production in the peripheral organs, particularly in the production of adrenal androgens. COU-AA-301, a multinational, randomized, double-blind phase III trial of abiraterone in patients with metastatic CRPC after docetaxel therapy, demonstrated a 3.9-month survival benefit of abiraterone/prednisone over placebo/prednisone. The subsequent COU-AA-302 trial established abiraterone’s role in the pre-chemotherapy space for CRPC, demonstrating a 4.4-month survival benefit [36, 62, 63]. However, as mentioned previously, approximately one-third of all patients had primary resistance to abiraterone use, and all patients with initial response eventually progressed by 15 months [36].

**Fig. 2:**
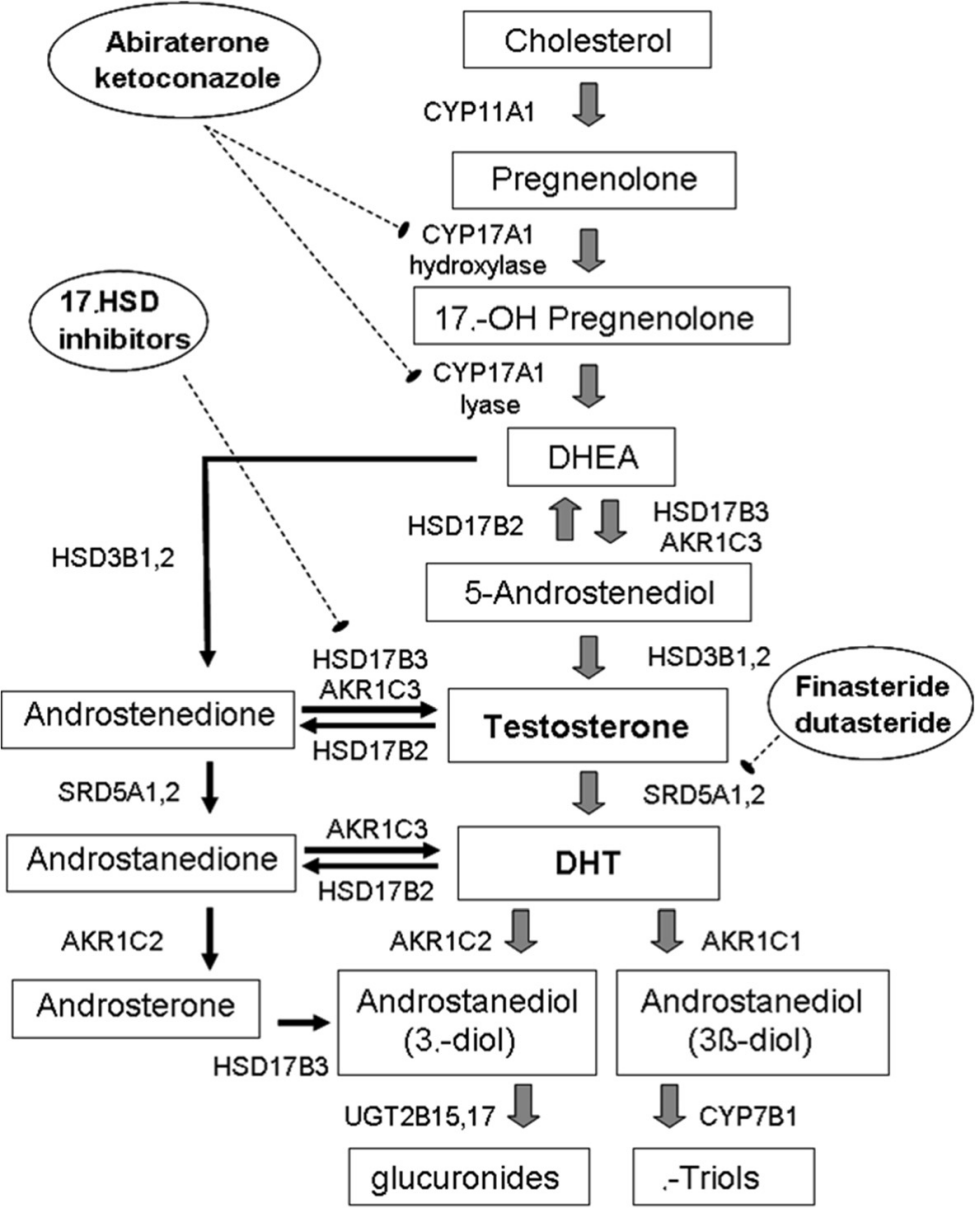
Androgen synthesis pathway. Reproduced with permission from Clinical Cancer Research [68]

Progression to CRPC includes utilization of the 5α-dione pathway, which allows prostate cancer cells to bypass testosterone in the steroidogenesis pathway (Fig. 2), leading to DHT production. However, CRPC cells are still dependent on adrenal androgens such as dihydroepiandrosterone and its sulfated form, which are converted to androstenedione in the prostate or adrenal gland by 3βHSD, an enzyme encoded by HSD3B. Androstenedione is then converted to DHT via a two-step process using 5α-androstenedione as an intermediary, with the enzymes 17βHSD3 and AKR1C3 (encoded by HSD17B3 and AKR1C3 respectively) mediating this conversion. By targeting adrenal androgen production, abiraterone prevents formation of adrenal androgen precursors needed for intratumoral androgen production [64].

As can be expected, however, patients who develop resistance to abiraterone demonstrate re-activation of intratumoral androgen production. Attard et al. [65] demonstrated that inhibition of CYP17A1 actually led to increased levels of the urinary metabolite 3α5α-17HP, which correlates with the excretion of androsterone, which in turn is the primary metabolite of 5α-reduced androgens such as DHT. The use of abiraterone may therefore push 17-hydroxyprogesterone towards the 5α-dione pathway.

Upregulation and mutations of the enzymes involved in the steroidogenesis pathway likely contribute to the progression to CRPC as well as resistance to abiraterone. Chang et al. [66] demonstrated that the 1245C mutation in HSD3B1, which was identified in treatment-naïve CRPC, was also identified in abiraterone-resistant xenograft models. Mostaghel et al. [67] demonstrated that abiraterone-treated LuCaP cell lines had a 1.3- to 4.5-fold increase in enzymes involved in the steroidogenesis pathway, including CYP17A1, AKR1C3, HSD17B3, and SDR5A2. Regulation of the steroidogenesis pathway is complex. Our group identified IL-6, which is upregulated in CRPC, as a mediator of increased expression of steroidogenic enzymes including HSD3B2 and AKR1C3, and IL-6 inhibition with small interfering RNA downregulated AKR1C3 expression [68]. AKR1C3 in particular is a very important enzyme in the steroidogenesis pathway, and its activation has been identified to contribute to CRPC resistance in patients treated with abiraterone and enzalutamide. There is a 16-fold increase of AKR1C3 in enzalutamide-resistant C42B cell lines [69]. Knockdown of AKR1C3 with shRNA or indomethacin, an AKR1C3 inhibitor, resensitized enzalutamide resistant cell lines to enzalutamide therapy [69].

### Enzalutamide

With the understanding that the androgen axis continues to be active and play an important role in the progression to CRPC, a new generation of AR signaling inhibitors are being developed. The best-studied agent in this class, and the only one approved for use, is enzalutamide (Xtandi, ENZA, MDV-3100). As opposed to first-generation anti-androgens, enzalutamide is an anti-androgen with multiple effects on AR – it is a competitive inhibitor of the C-terminus ligand-binding domain, but it also prevents AR nuclear translocation, AR binding to DNA, and co-activator recruitment [44]. The AFFIRM III trial, a phase III, double-blind, placebo-controlled trial in patients with CRPC who had failed docetaxel, demonstrated a 4.8-month survival benefit over placebo, and the subsequent PREVAIL trial demonstrated enzalutamide’s effectiveness in pre-chemotherapy CRPC patients [70, 71]. Just as in the trials evaluating abiraterone, there is a subset of patients who have primary resistance to enzalutamide therapy – in the AFFIRM trial, approximately 25 % of patients progressed within the first 3 months of therapy. By 24 months, all patients had progressed on enzalutamide [71].

Our lab has focused extensively on the process of autophagy, one potentially important physiologic process that may contribute to resistance to many therapies, including enzalutamide, and may be an important adjunctive target for treatment. Autophagy is a physiologic catabolic process that is constitutively active at a basal rate, but can be activated in response to stressors. When activated, it utilizes lysosomal-mediated degradation of cellular proteins and organelles to regenerate energy [72–75]. Cancer cells can activate autophagy to prolong survival under harsh conditions of metabolic stress induced by various therapeutic modalities, but if pushed to excessive or deregulated autophagy, this process can induce type-II programmed cell death [76, 77]. ADT has been demonstrated to induce autophagy, though the exact mechanism is not yet known [78]. Inhibiting autophagy is a potential target for adjunctive therapy, as we will discuss later.

As enzalutamide also targets the ligand-binding domain of AR, point mutations in this region can lead to secondary resistance as well. The Phe876Leu mutation has been reported to make enzalutamide act more as an agonist than an antagonist, but this has not yet been clinically documented [79, 80]. Similar effects were noted for the first-generation anti-androgens, so it can be inferred that the same process is applicable for enzalutamide as well.

Another proposed mechanism is the “glucocorticoid receptor take-over” pathway. Glucocorticoid receptors are nuclear receptors similar in structure to AR, and glucocorticoids initially have a suppressive effect on prostate cancer; they are often given in conjunction with early treatments of CRPC. However, since the DNA binding domain (DNB) of the glucocorticoid receptor is very similar to the DBD of AR [81, 82], and the glucocorticoid receptor has been shown to bind to many AR regulated genes, its upregulation in patients treated with chemotherapy or ADT may contribute to enzalutamide resistance [83].

## Androgen receptor splice variants (ARVs)

ARVs are truncated versions of the wild type AR that are constitutively active. The truncated portion is typically the C-terminal ligand-binding domain [84–87], though at least one variant, ARV8, was reported to have loss of the DNA binding domain [88]. The loss of the LBD makes these variants ligand-independent. The true functional implication of ARVs is not yet completely understood, as direct measurement of the variants has been limited by lack of variant-specific antibodies, requiring proxy assessment using transcribed RNA levels. However, transcribed RNA levels may not be reflective of protein levels, suggesting some degree of post-translational modification [85, 86].

The role of ARVs in clinical CRPC is being established however. While some CRPC cell lines demonstrate low levels of ARVs, CWR22Rv1 in particular demonstrates almost equal levels of ARV and full-length AR [17]. Hornberg et al. [89] demonstrated that there were higher levels of ARV expression in CRPC bone metastases compared to hormone-sensitive prostate cancer bone metastases, and that ARV expression was associated with poorer prognosis.

Research in our lab, as well as others [90–92], strongly support the role of ARV’s as mechanisms of resistance in CRPC. As can be expected, the loss of the ligand-binding domain removes the target of androgen-signaling inhibitors such as enzalutamide, and CRPC is able to overcome the loss of intratumoral androgens mediated by abiraterone and anti-steroidogenesis agents. Li et al. [91] demonstrated that knockdown of ARV7 in CWR22Rv1 cells restored responsiveness to anti-androgens, which makes this an important target for future therapies. The clinical significance of this is underscored by the findings of Antonarakis et al. [93], who demonstrated that the presence of ARV7 in circulating tumor cells in patients treated with enzalutamide or abiraterone had a significantly lower prostate-specific antigen (PSA) response, shorter progression-free survival, and shorter overall survival compared to men without ARV7. This is supported in the more recent work by Azad et al. [94], in which pre-treatment AR gene aberration (copy number increase and/or exon 8 deletion) on copy-free DNA was predictive of poorer PSA response and shorter time to radiographic/clinical progression. Indeed, this may contribute to the primary resistance to enzalutamide and abiraterone in the AFFIRM III and COU-AA-301 trials, respectively. Interestingly, in recent data presented by Antonarakis et al. [95], there was no significant difference in PSA response or progression-free survival in patients treated with docetaxel regardless of ARV7 presence in circulating tumor cells. When comparing the abiraterone and enzalutamide treated patients, the ARV7-positive subset treated with docetaxel had better PSA response and longer median progression-free survival [95]. This suggests that taxanes may be less susceptible to primary resistance in ARV7-positive patients, and therefore may be a better option for initial treatment in patients with known ARV7 expression.

## Emerging strategies

These various mechanisms of resistance to currently approved therapies of CRPC are each potential targets for new therapies. Below, we focus on the emerging strategies for identifying new management options.

Our focus on autophagy has led to the identification and assessment of various adjunctive medical therapies. By utilizing autophagy inhibitors, such as clomipramine and metformin, our group and others have demonstrated effective cytotoxic results either as monotherapy or in conjunction with known therapeutic agents. Specifically, in the setting of CRPC, we demonstrated that clomipramine and metformin significantly increased cytotoxicity in *in vitro* and *in vivo* mouse models when used in conjunction with enzalutamide – the enzalutamide/clomipramine combination decreased tumor volume by 91 %, the enzalutamide/metformin combination decreased it by 78 %, while enzalutamide alone caused a 25–50 % decrease [74]. Ongoing clinical trials are exploring the adjunctive role of metformin with enzalutamide therapy in CRPC patients.

Another important target is the NTD of the AR, which has less than 15 % homology with the NTD of other steroid receptors [96–98] and is important for transactivation. As a preserved portion of the splice variant as well as full-length AR, it has promise as a target for therapy to address ligand-independent androgen axis activation. EPI-001, which is a small molecular NTD inhibitor, has been evaluated by Sadar et al. [99, 100]. It functioned as an effective and specific inhibitor of AR transcriptional activity, even in the presence of increasing androgen levels. As it targets the NTD, it was effective at attenuating ARV activity *in vitro* and *in vivo* [100]. Along similar lines, AR degradation enhancers, such as ASC-J9 developed by Chang et al. [101], target both full-length AR and ARVs.

Our group has turned its attention to specific inhibitors of the splice variants. Niclosamide, a currently approved anti-helminthic medication, was identified as an inhibitor of ARV7 activity via multiple mechanisms. It inhibited ARV7 transcriptional activity where enzalutamide did not; it did so by reducing ARV7 recruitment to the promoter regions of downstream targets. It also inhibits ARV7-specific protein expression and increases protein degradation. MG132, a 26S proteasome inhibitor, reduced niclosamide-mediated inhibition of ARV7 protein expression, suggesting that niclosamide induced ARV degradation via a proteasome-dependent pathway. In enzalutamide-resistant C42B cells expressing ARV7, niclosamide was noted to have significant dose-dependent cytotoxic effects. When used in conjunction with enzalutamide, it demonstrated an additive response [102].

New techniques for identification of therapeutic agents are also being developed. Drug-seq, a modification of the ChIP-seq technology, utilizes a genome-wide binding screen of a potential therapeutic agent in various physiologic conditions to identify potential therapeutic benefits. In this manner, SD-70, a synthetic chemical in a molecular library that was identified as an inhibitor of prostate cancer translocation events, was found to co-localize with AR-bound functional enhancers in a DHT-dependent manner. Functionally, it was determined to be a histone demethylase inhibitor, with one of its targets being KDM4C. It has been identified to have *in vitro* cytotoxic effects on hormone-sensitive LNCaP cells, C42B and drug-resistant C42B cells, and *in vivo* efficacy in a CWR22Rv1 mouse xenograft model [103].

In addition, there continues to be development of agents that target the AR axis. These agents are briefly summarized in Table 1 [104, 105]. They may have a role in the treatment of CRPC either as monotherapy or in conjunction with some of the newer targets described.

**Table 1:** AR axis targeting drugs in clinical development

Agent	Pharmaceutical company	Mechanisms of action/target	Current development status
ARN-509	Johnson & Johnson	AR antagonist – inhibits nuclear transportation, inhibits DNA binding	In multiple phase III clinical trials (SPARTAN, etc.)
AZD3514	Astra Zeneca	Small molecule modulating AR through two distinct mechanisms	Completed Phase I recruitment
EPI-001	ESSA Pharma Inc.	Inhibiting the N-terminus of the AR protein	Awaiting clinical development
ODM-201	Bayer HealthCare	AR antagonist – distinct from enzalutamide and ARN509	In phase III clinical trial (ARAMIS)
OGX-011 (Custirsen)	OncoGenex Pharmaceuticals and Teva Pharmaceuticals	Second-generation antisense drug that targets clusterin, a secreted protein that acts as a cell-survival protein and is over-expressed in response to anti-cancer agents	In phase III clinical trials (AFFINITY)
OGX427 (Apatorsen)	OncoGenex Pharmaceuticals	Second-generation antisense drug targeting HSP27	In phase II clinical trials
TAK-700 (Orteronel)	Takeda Pharmaceutical Company	Non-steroidal imidazole inhibitor of CYP17A1	In phase III clinical trial
TOK-001 (Galeterone)	Tokai Pharmaceuticals	CYP17 lyase inhibitor Competitive AR antagonist (binding to the steroid-binding pocket of AR)	Phase III randomized control trial (ARMOR3 trial)
VT-464	Viamet Pharmaceuticals	Non-steroidal CYP17 lyase inhibitor AR antagonist activity independent of CYP17 lyase inhibition	Phase II clinical trial

## Cross-resistance and sequencing of therapies

As more treatments become approved for CRPC, sequencing these treatments becomes more problematic. Cross-resistance has become evident, limiting use of these agents in patients who have failed prior therapy. Cross-resistance is unfortunately not limited to any one class of agents, but rather seems to involve all the approved therapies for CRPC.

Cheng et al. [106] demonstrated in a large retrospective study of 310 patients with metastatic CRPC patients that prior abiraterone or docetaxel treatment blunted subsequent enzalutamide response, confirming findings from multiple smaller studies. PSA decline and PSA progression-free survival were both significantly blunted in patients who had been previously treated with abiraterone, and less so in patients with prior docetaxel therapy [106]. Other studies demonstrated similar findings in men treated with docetaxel following abiraterone [107, 108], and Nadal et al. [109] confirmed blunted efficacy of enzalutamide in docetaxel-treated patients. Taxane efficacy following AR targeted therapy is also blunted, as demonstrated by van Soest et al. [110, 111] – docetaxel inhibited tumor growth, AR nuclear translocation, AR regulated gene expression, and PSA levels in enzalutamide-naïve tumors in castrated mice but not in enzalutamide-resistant tumors. This cross-resistance suggests that taxane therapy does in fact have a role in AR axis modulation, inhibiting AR trafficking via microtubules [112]. Importantly, however, the cross-resistance occurs regardless of the sequencing of docetaxel and AR targeted therapies.

Cross-resistance with AR targeted therapies and cabazitaxel appears to be less significant. As previously mentioned, cabazitaxel was developed to overcome the resistance to docetaxel mediated via P-glycoprotein [60]. It was subsequently identified to have unique mechanisms of actions compared to docetaxel [59], which may account for why it does not have the same cross-resistance with AR targeted therapies. van Soest et al. [110] concurrently evaluated cabazitaxel efficacy in enzalutamide-naïve and enzalutamide-resistant tumors in castrated mice and found that cabazitaxel remained highly effective in enzalutamide-resistant tumors, and more importantly, was much more potent than docetaxel independent of the AR pathway. Al Nakouzi et al. [113] confirmed similar findings *in vivo* and *in vitro*. As a result, an ongoing clinical trial is evaluating the role of cabazitaxel in chemotherapy-naïve CRPC (FIRSTANA).

Cross-resistance has also pushed forward the need to identify molecules that can inhibit resistance pathways and emphasized the role of combination therapy. The CHAARTED trial demonstrated the strength of combination therapy by providing the largest survival benefit of any treatment regimen in advanced prostate cancer, by treating patients with docetaxel and ADT [40, 114].

The agents identified above, such as the autophagy inhibitors metformin and clomipramine, the ARV7 inhibitor niclosamide, the NTD inhibitor EPI-001, the AR degradation promoter ASC-J9, and novel agents such as SD70, are all important adjuncts to currently approved therapy. However, their efficacy may be compounded by utilization in conjunction with approved therapies rather than as competitive agents. SD70, as we demonstrated, had additive cytotoxic effect when used with enzalutamide, abiraterone, and docetaxel. As such, the future likely lies in novel combination therapies rather than monotherapies.

## Conclusions

CRPC is an incurable cancer characterized by progression despite multiple currently approved therapies. By understanding the mechanisms of resistance to currently approved treatments, targeted therapies can help overcome these resistance pathways and provided clinical gains in the treatment of this patient population. Combination therapy may be the next advancement in the treatment of CRPC.

## Abbreviations

ABCB1: ATP-binding cassette transporter family
ADT: Androgen-deprivation therapy
AR: Androgen receptor
ARV: Androgen-receptor variant
CRPC: Castration-resistant prostate cancer
DHT: Dihydrotestosterone
LBD: Ligand-binding domain
NTD: N-terminal domain
PSA: Prostate-specific antigen
